# Early Postpartum Stress, Anxiety, Depression, and Resilience Development among Danish First-Time Mothers before and during First-Wave COVID-19 Pandemic

**DOI:** 10.3390/ijerph182211734

**Published:** 2021-11-09

**Authors:** Monica Ladekarl, Nanna Julie Olsen, Karoline Winckler, Anne Brødsgaard, Ellen Aagaard Nøhr, Berit Lilienthal Heitmann, Ina Olmer Specht

**Affiliations:** 1 Research Unit for Dietary Studies, The Parker Institute, Bispebjerg and Frederiksberg Hospital, 2000 Frederiksberg, Denmark; Nanna.Julie.Olsen@regionh.dk (N.J.O.); karoline.winckler@regionh.dk (K.W.); Berit.Lilienthal.Heitmann@regionh.dk (B.L.H.); Ina.Olmer.Specht@regionh.dk (I.O.S.); 2 Department of Gynaecology and Obstetrics, Copenhagen University Hospital Amager Hvidovre, 2650 Hvidovre, Denmark; Anne.Broedsgaard.Madsen@regionh.dk; 3 Department of Paediatrics and Adolescent Medicine, Copenhagen University Hospital Amager Hvidovre, 2650 Hvidovre, Denmark; 4 Department of Public Health-Nursing and Health Care, Aarhus University, 8000 Aarhus, Denmark; 5 Research Unit for Gynaecology and Obstetrics, Department of Clinical Research, University of Southern Denmark, 5000 Odense C, Denmark; eanohr@health.sdu.dk; 6 The Boden Institute of Obesity, Nutrition, Exercise & Eating Disorders, The University of Sydney, Camperdown 2006, Australia; 7 Section for General Practice, Department of Public Health, University of Copenhagen, 1014 København K, Denmark

**Keywords:** COVID-19, lockdown, mental health, motherhood, post-partum, resilience

## Abstract

On 11 March 2020, a lockdown to limit the spread of COVID-19 was implemented in Denmark. The pandemic and the lockdown might have caused stress, depression, and anxiety in new mothers. Individuals with high resilience to stress may have been less affected. This study aimed to investigate if changes in perceived stress, anxiety, depression, and resilience from the second trimester until two months postpartum were different before and during the COVID-19 pandemic in Denmark in spring 2020. Pregnant women enrolled in an ongoing feasibility study completed an online questionnaire measuring perceived stress, depression, anxiety, and resilience in the second trimester and two months postpartum. Changes in scores between women completing the two-month postpartum questionnaire before (*n* = 26) or during (*n* = 47) the COVID-19 pandemic were calculated. No statistically significant differences in changes from baseline to follow-up between pre- and during-pandemic groups in Cohen’s Perceived Stress Scale (PSS), the Depression, Anxiety, Stress Scale (DASS), or the Connor–Davidson Resilience Scale (CD-RISC) were found. Adjusted differences in group means were as follows: PSS: 0.70 (CI—2.45; 3.85); DASS Stress: 0.76 (CI—3.59; 2.08); DASS Anxiety: 0.47 (CI—0.84; 1.77); DASS Depression: 0.88 (CI—0.95; 2.71); and CD-RISC: 1.19 (CI—3.16; 5.54). In conclusion, we did not find significant differences in the development of stress, depression, anxiety, or resilience before or during the Danish COVID-19 pandemic in spring 2020.

## 1. Introduction

A coronavirus disease (COVID-19) pandemic was declared on 11 March 2020 by the World Health Organization [1]. The same day, a lockdown to constrain and limit the spread of COVID-19 was implemented in Denmark. The pandemic and the lockdown might have caused stress, depression, and anxiety in both new mothers and the general population. The lockdown included, among other things, a ban of physical attendance at most workplaces without critical health and medical care function; closure of universities, schools, pre-schools, nurseries, restaurants, cultural institutions, and fitness centers; and restriction of public transport. In the healthcare sector, non-acute procedures and examinations were postponed or transformed to video or phone consultations. The delivery ward discharged all low-risk families 3–6 h after birth in the first three weeks of April 2020, because the maternity ward was reserved for potential COVID-19 patients, but the postpartum home visits offered by public health nurses were continued with a few restrictions [2]. Schools up to fifth grade were reopened 15 April 2020, while most of the remaining measures were gradually lifted in May and June 2020 [3].

The early postpartum period is a critical period where maternal stress may impact the health and emotional development of the newborn. A recent study found that a high number of postpartum stressful events was associated with lower cortisol response in the infant and that lower stress reactivity in the infant was associated with higher externalizing symptoms at 12 months of age [4]. In addition, maternal stress in the early postpartum period has been found to be associated with excessive crying, feeding problems, and sleep problems in infants [5,6,7]. Stress in the early postpartum period may also impact the mother’s health, including greater postpartum weight retention [8].

A few other studies have reported maternal mental wellbeing development from gestation until postpartum under the COVID-19 pandemic. A Japanese study of 280 women with routine health checkups one month after birth performed either one year before the COVID-19 pandemic (n = 148) or during the COVID-19 pandemic (n = 132) compared the two group’s scores using the Edinburgh Postnatal Depression Scale (EPDS) and the Japanese version of the Mother-to-Infant Bonding Scale (MIBS-J). They found that the depression scores were similar for the two groups, while the MIBS-J was higher in the COVID-19 group [9]. A cross-sectional online survey of 5866 Belgian women, 2421 pregnant and 3445 postpartum, carried out a few weeks after the lockdown, found that 23.6% of the breastfeeding women had depressive symptoms according to EPDS and 42.4% had anxiety symptoms [10]. A Turkish study in low-risk women conducted two days postpartum at a maternity ward in June 2020 (*n* = 223) found doubled EPDS depression scores compared to a similar study made in 2016–2017 [11]. An American mixed-methods study of 16 pregnant and 15 postpartum women reported six times higher levels of prenatal anxiety during the pandemic as compared to previous studies conducted in the Colorado area, and wellbeing and resilience scores were lower than before the pandemic [12]. Another American cross-sectional study including 885 women found that participants had higher stress and lower resilience relative to pre-pandemic numbers, and that resilience and mastery were related to lower stress, depression, and anxiety [13]. An Italian cross-sectional study of 163 women found depressive symptoms in 44.2% (using EPDS) of the participants and post-traumatic stress syndrome (PTSS) in 42.9% of them.

Previous studies have shown reduced stress in people with high resilience [14,15], suggesting that resilient individuals are more resistant to stressors. Resilience is defined as the successful adaptation to adversities, including successful recovery from adverse life events and sustainability in relation to life challenges [16]. In this article, we define “stress” as the subjective feeling of inner tension assessed by the Perceived Stress Scale [17], while “resilience” refers to the individual’s perception of their ability to bounce back from adversity and is measured by the Connor–Davidson Resilience Scale [18]. In short, “stress is when an individual perceives that environmental demands exceed his or her adaptive capacity” [19].

The aim of this study was to investigate if changes in perceived stress, anxiety, depression, and resilience from early pregnancy until two months postpartum were different among women examined in the months before and during the Danish COVID-19 spring lockdown effectuated on 11 March 2020.

## 2. Materials and Methods

Between 18 June and 22 November 2019, we recruited 124 healthy pregnant women expecting their first child and assigned to antenatal care and birth at Copenhagen University Hospital, Amager Hvidovre in Denmark, to participate in a randomized feasibility study, the OBEAT—Beating Obesity RCT.

The obstetric department has approximately 7000 births a year. Women attending antenatal care at the hospital are healthy women, as well as women with medical and obstetric complications and women with psychosocial problems. Access to universal healthcare in Denmark is free of charge, and all pregnant women are offered public antenatal care delivered by hospital-employed midwives.

The OBEAT intervention aimed at preventing excess gestational and postpartum weight gain and overweight development in the offspring by using a digital resilience-building program in the intervention group to improve stress management during pregnancy. This program is a flexible psychoeducation program that we specifically tailored toward pregnant women, and it was developed by the Danish Committee for Health Education [20]. The program was accessible through links to a homepage sent by mail every week to the intervention group. It consisted of 19 different modules based on research on mentalization, mindfulness, parent management training, improving self-control, self-efficacy, cognitive behavior therapy, and social learning theory. These techniques have been found to be effective in improving engagement in health behaviors and reducing symptoms and negative behaviors in clinical groups [21]. The control group received standard care.

The women were recruited at their first antenatal midwife visit. All pregnant women fulfilling the inclusion criteria could participate in the OBEAT study and were sent information about the study to their personal and secure digital mailbox “e-Boks” linked to their unique personal identification number together with the invitation to their first appointment. After that, the research team reinformed them at the midwifery center, and there were posters and information leaflets available in the waiting areas.

Exclusion criteria were non-Danish speaking, obesity (BMI > 30 kg/m^2^), type I or type II diabetes, 18–50 years of age, and multiple pregnancies. In addition, women with any psychiatric diagnoses or severe social problems were excluded.

**Figure 1:**
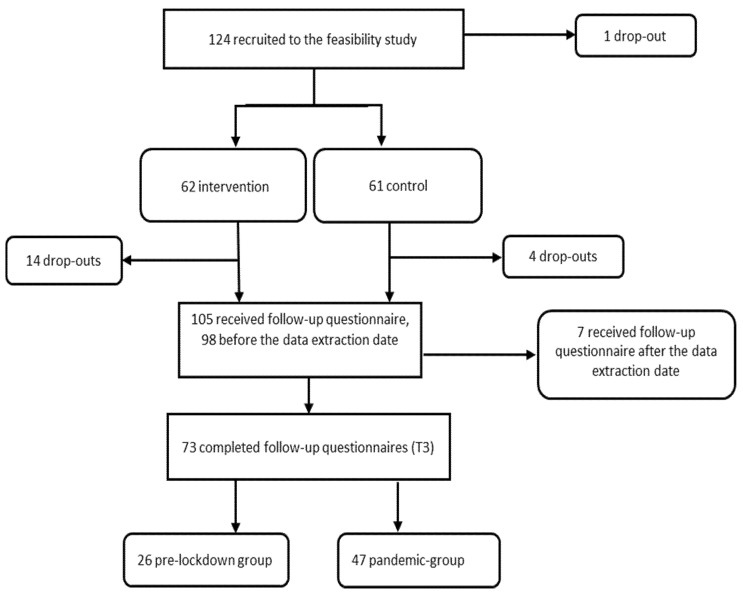
Flowchart.

All women were asked to complete an online questionnaire at four time points: baseline at gestational weeks 14–20 (T0), gestational weeks 28 (T1) and 35 (T2), and follow-up at two months postpartum (T3). A total of 98 participants received the T3 questionnaire (19 participants had left the study and 7 participants were not yet two months postpartum at the data extraction date), and 73 (75%) completed it between 13 January and 4 June 2020. Thus, 26 participants completed the online questionnaire at T3 between 13 January and 11 March 2020 (pre-lockdown-group), while 47 completed the online questionnaire at T3 between 12 March and 4 June 2020 (pandemic-group). A flowchart of the study is shown in Figure 1.

### 2.1. Outcomes

We used the following scales to measure mental health and resilience at all four time points:

Cohen’s Perceived Stress Scale (PSS): PSS measures the respondent’s experience of stress over the past four weeks using ten questions to answer to what extent the respondent experiences his life as unpredictable, uncontrollable, and stressful, and whether they feel nervous or stressed. The scale is from 0 to 40. The higher the score, the higher the stress level experienced [17]. The test is not diagnostic and was initially intended as a continuous score, but some authors have suggested the following categories for low, moderate, and severe stress [22]: scores ranging from 0–13 are considered low stress; scores ranging from 14–26 are considered moderate stress; and scores ranging from 27–40 are considered high perceived stress.

The Depression Anxiety Stress Scales (DASS): DASS is a 42-item self-report instrument designed to measure the three related negative emotional states of depression, anxiety, and stress. The scale is from 0 to 42 for every emotional state. The higher the score, the higher the level of depression, anxiety, or stress experienced [23]. Ranges are as follows: Stress: Normal—0–10; Mild—11–18; Moderate—19–26; Severe—27–34; Extremely severe—35–42. Anxiety: Normal—0–6; Mild—7–9; Moderate—10–14; Severe—15–19; Extremely severe—20–42. Depression: Normal—0–9; Mild—10–12; Moderate—13–20; Severe—21–27; Extremely severe—28–42.

Resilience was measured using the Connor–Davidson resilience scale (CD-RISC). The scale comprises 25 items, each rated on a 5-point scale (0–4). Thus, the total score ranges from 0–100, with higher scores reflecting greater resilience [18].

The Danish versions of the PSS and CD-RISC inventories were both validated [24,25]. DASS was translated to Danish by Dr. Mikael Thastum from the University of Aarhus [26] and has previously shown good psychometric properties [27].

The used instruments were selected to measure different traits of psychological stress and emotion regulation; while PSS measures experiences of non-specific stress, the items forming the stress scale of DASS measure symptoms that reflect chronic non-specific arousal [28]. In addition, DASS measures symptoms reflecting depression and anxiety, while CD-RISC reflects the perceived level of resilience to stress.

In addition to the abovementioned instruments, the women also self-reported on diet, stressful life events, physical activity, weight, sleep, demographic information, and pregnancy complications in the four questionnaires.

### 2.2. Statistical Methods

Characteristics of the pre-lockdown group and the pandemic group are presented as number (n) and percentage (%) for categorical variables and mean and standard deviation (SD) for continuous variables. Changes in PSS, DASS, and CD-RISC scores between baseline and follow-up were separately calculated for each woman within the groups of women completing the two-month postpartum questionnaire before (*n* = 26) and during (*n* = 47) the mandatory COVID-19 lockdown. Analyses were performed using two-sided analysis of variance, adjusted for allocation group and baseline values of the outcome. In addition, sensitivity analyses of baseline PSS, DASS, and CD-RISC scores among women with missing information at follow-up (*n* = 18) and completers (*n* = 73) were conducted.

## 3. Results

Sample characteristics are shown in Table 1. The majority of women within both the pre-lockdown and pandemic groups had a high educational level (53.9% and 51.1%, respectively), with a mean age of 30.2 and 30.7 years at baseline, respectively. The groups differed according to group allocation; 50.0% (*n* = 13) of the women in the pre-lockdown group were randomized to the intervention group compared to 38.3% (*n* = 18) of the women in the pandemic group. Women within both groups had low/normal PSS, DASS, and CD-RISC scores at baseline. Changes in PSS, DASS, and CD-RISC from baseline to follow-up did not differ between women who answered the postpartum questionnaires pre- or during the pandemic (Table 2). Sensitivity analyses showed that women that did not complete the questionnaire at follow-up had a 3.95 higher score in the Depression subscale of DASS at baseline (*p* = 0.05) than that of women who completed the follow-up. Otherwise, women that did not complete the questionnaire at follow-up did not differ in the outcome variables.

## 4. Discussion

The COVID-19 pandemic could be seen as a potent psychosocial stressor because of increased known risk factors for psychological distress, i.e., fear of disease, economic uncertainty, social isolation, and changed healthcare services [29]. However, in our study, we did not find evidence of significant differences in the development of stress, depression, anxiety, or resilience in women from week 14–20 in pregnancy until two months postpartum between women experiencing the Danish lockdown in the spring of 2020 and those who did not. Other cross-sectional studies giving early accounts of the effect of the early COVID-19 pandemic on mental wellbeing in the general population have tended to show higher stress, depression, and anxiety levels, even if the results are somewhat mixed due to differences in methods, populations, location, and timing regarding the pandemic’s course [29]. In contrast to this, but in accordance with our results, an Israeli study of 346 women found a lower risk of postpartum depression during the COVID-19 strict isolation period than before the pandemic [30]. There could be several explanations for why our results differ from most of the previous studies. The Danish lockdown was introduced on 11 March 2020, 14 days after the first Danish COVID-19 case and at a time when only ten individuals had been hospitalized. After the lockdown, the number of infected cases in Denmark came under control very fast. The hospitals were never overwhelmed, and the governmental financial help packages secured financial stability. Access to universal healthcare in Denmark is free of charge and is not dependent on job status, as in many insurance-based healthcare systems. All of these factors may have contributed to reducing the general insecurity in the society, including the insecurity felt by new mothers. This notion is supported by a multinational survey reporting that Denmark scored high in satisfaction with how the country had handled the COVID-19 pandemic. Moreover, most Danes (73%) thought that the outbreak had not significantly changed their everyday life [31]. While the pandemic may have caused concerns about the seriousness of disease for the fetus and the infant, the restrictions in healthcare services and family support, other factors caused by the pandemic may have had a beneficial impact on mental wellbeing [29], and may explain our observations of unchanged stress scores. Examples include having the support of a partner working from home for a longer period or a reduction in social pressure, i.e., no visits on the maternity ward and no public support groups for new mothers.

A limitation to our study is that the participants were not representative of the general population. In general, the women in our sample were older and better educated than the average Danish pregnant woman. We also only included Danish-speaking women. Our results might have been different in socially more disadvantaged women with partners who may be more likely to experience job insecurity or have jobs where they were more exposed to the disease, i.e., working as a bus driver or at a meat factory. On the other hand, an Italian study showed that a higher educational status was associated with increased prevalence of anxiety during the COVID-19 pandemic, challenging this explanation [32].

Another significant limitation of our study was the relatively small sample size of 73 women. Because the data were collected as a part of another study, we did not conduct a formal sample size calculation in advance. It is possible that our sample was underpowered to detect a difference when the effect size was not large enough, leading to a possible type 2 error in the findings. Using data for another purpose than the original intention can create bias, but the COVID-19 pandemic and the subsequent consequences of the imposed lockdown were a very extraordinary situation, so all relevant knowledge gained from it should be disseminated. Furthermore, there were more women from the intervention group in the pre-lockdown group than in the pandemic group. The intervention was designed to build resilience, and even though the program’s effect is still unknown, it might have made the pre-lockdown group less prone to stress. This might have affected the results, even though we adjusted for the allocation group in our analysis. Although the PSS, DASS, and CD-RISC instruments have separate scores and psychometric properties, it cannot be ruled out that they may have some concurrent overlapping, which could potentially introduce some misclassification. The magnitude and direction of this bias are considered challenging to estimate, and such misclassification could potentially lead to either over- or under-estimating the observed results. In addition, the PSS scale measures stress experienced in the past 4 weeks; 15 out of the 47 women in the pandemic group received the follow-up questionnaire during the four weeks after the lockdown. Therefore, we do not know if the answers provided by these women could in part have reflected some of their stress related to the periods before the lockdown. Finally, the questionnaires were part of an already ongoing study, and there were no questions explicitly aimed at concerns about COVID-19. Thus, COVID-19-specific worries were not measured.

An important strength in our study is that we were able to compare prospective differences in changes from baseline scores to 2 months postpartum instead of cross-sectional analyses of a score obtained at a single time point. Thus, we had access to a range of possible confounders and demographic information and were able to test the two groups’ comparability. Furthermore, the follow-up data were collected during a relatively short period of 5 months, and, unlike other studies [33], the women were all recruited the same way, at their first antenatal visit at the midwife. Finally, all participants received the questionnaires at fixed time points during pregnancy and postpartum, and we used validated instruments to measure depression, anxiety, resilience, and stress.

## 5. Conclusions

In conclusion, this study showed no differences in changes in PSS, DASS scales, or CD-RISC scores from the second trimester to 2 months postpartum between women with follow-up measures before or during the COVID-19 lockdown in Denmark. These results differed from results reported in studies focusing on new mothers in other countries, potentially due to the higher level of satisfaction in the Danish population about the political handling of the lockdown situation in Denmark.

## Figures and Tables

**Table 1:** Study participant characteristics divided into pre-lockdown and during pandemic groups.

	Pre-Lockdown Group (*n* = 26)			Pandemic Group (*n* = 47)		
	%	Mean	SD	%	Mean	SD
Allocation group						
Intervention	50.0			38.3		
Control	50.0			61.7		
Civil status						
Living with partner	96.1			100		
Single	3.9			0		
Education						
High school or less	11.5			10.6		
Low up to 3 y	3.9			4.3		
Medium, 3–5 y	30.8			34.0		
High > 5	53.9			51.1		
Age (years)		30.2	3.9		30.7	3.4
Gestational age (weeks)		40.2	1.3		40.0	1.2
Birth weight (g)		3596	529		3453	410
Pre-pregnancy BMI (kg/m^2^)		23.4	2.7		23.2	2.8
PSS T0 (Score)		10.7	5.8		12.1	4.5
DASS T0 Stress (Score)		6.0	5.3		7.0	5.6
DASS T0 Anxiety (Score)		2.7	3.3		2.7	2.8
DASS T0 Depression (Score)		1.4	1.7		2.2	2.2
CD-RISC T0 (Score)		95.7	7.8		96.3	9.9

PSS: Perceived Stress Scale. Higher scores (max. 40) indicate higher perceived stress. DASS: Depression, Anxiety, Stress Scale. Higher scores (max. 28 each) indicate greater severity of stress, anxiety, and depression. CD-RISC: the Connor–Davidson Resilience Scale. Higher scores (max. 100) indicate higher perceived resilience.

**Table 2:** Changes in PSS, DASS, and CD-RISC from baseline to follow-up among women completing follow-up pre-lockdown or during COVID-19 pandemic, adjusted for baseline level and allocation group.

	Pre-Lockdown (*n* = 26)		Pandemic Group (*n* = 47)		Differences between Pre-Lockdown and Pandemic Group, Crude		Differences between Pre-Lockdown and Pandemic Group, Adjusted		
	Change from baseline to follow-up, adjusted	95% CI	Change from baseline to follow-up, adjusted	95% CI	Difference, group means	95% CI	Difference, group means	95% CI	*p*-value
PSS	1.12	−1.39; 3.62	1.82	−0.07; 3.71	−0.43	−4.1; 3.3	0.70	−2.45; 3.85	0.66
DASS Stress	1.85	−0.40; 4.11	1.10	−0.62; 2.81	1.16	−4.2; 1.8	−0.76	−3.59; 2.08	0.60
DASS Anxiety	−0.99	−2.03; 0.04	−0.52	−1.31; 0.27	0.56	−0.98; 2.11	0.47	−0.84; 1.77	0.48
DASS Depression	0.29	−1.15; 1.74	1.17	0.08; 2.27	0.59	−1.2; 2.4	0.88	−0.95; 2.71	0.34
CD-RISC	0.36	−3.12; 3.83	1.55	−1.04; 4.14	0.89	−3.8; 5.6	1.19	−3.16; 5.54	0.59

PSS: Perceived Stress Scale. A higher score (max. 40) indicates higher perceived stress. DASS: Depression, Anxiety, Stress Scale. Higher scores (max. 28 each) indicate higher levels of stress, anxiety, and depression.CD-RISC: the Connor–Davidson Resilience Scale. A higher score (max. 100) indicates higher perceived resilience.

## Data Availability

Data from the OBEAT intervention contains sensitive information and cannot be made publicly available for ethical and legal reasons. Public availability may compromise participant privacy, and this would not comply with Danish legislation (www.datatilsynet.dk, accessed on 28 September 2021). Access to the data requires an application submitted to and subsequently approved by the steering committee. Data requests may be sent to Professor Berit L. Heitmann (Berit.Lilienthal.Heitmann@regionh.dk) or the Research Unit for Dietary Studies at The Parker Institute (bfh-eek@regionh.dk).
